# Impact of glycaemic control on complication rates after breast reconstruction

**DOI:** 10.1093/bjsopen/zrag012

**Published:** 2026-03-09

**Authors:** Samuel A Knoedler, Victoria Kong, Julius M Wirtz, Leonard Knoedler, Adriana C Panayi, Thomas Schaschinger, Amir K Bigdeli, Gabriel Hundeshagen, Omar Allam, Fortunay Diatta, Martin Kauke-Navarro

**Affiliations:** Department of Plastic Surgery and Hand Surgery, Klinikum Rechts der Isar, Technical University of Munich, Munich, Germany; Division of Plastic Surgery, Department of Surgery, Yale School of Medicine, New Haven, Connecticut, USA; Division of Plastic Surgery, Department of Surgery, Yale School of Medicine, New Haven, Connecticut, USA; UCSF Medical Center, University of California San Francisco, San Francisco, CA, USA; Division of Plastic and Reconstructive Surgery, Cedars-Sinai Medical Center, Los Angeles, CA, USA; Department of Hand-, Plastic and Reconstructive Surgery, Microsurgery, Burn Trauma Center, BG Trauma Center Ludwigshafen, University of Heidelberg, Ludwigshafen, Germany; Department of Hand-, Plastic and Reconstructive Surgery, Microsurgery, Burn Trauma Center, BG Trauma Center Ludwigshafen, University of Heidelberg, Ludwigshafen, Germany; Department of Plastic, Reconstructive, Aesthetic and Hand Surgery, Klinikum Kassel, Teaching Hospital of Philipps University Marburg, Kassel, Germany; Department of Hand-, Plastic and Reconstructive Surgery, Microsurgery, Burn Trauma Center, BG Trauma Center Ludwigshafen, University of Heidelberg, Ludwigshafen, Germany; Division of Plastic Surgery, Department of Surgery, Yale School of Medicine, New Haven, Connecticut, USA; Division of Plastic Surgery, Department of Surgery, Yale School of Medicine, New Haven, Connecticut, USA; Division of Plastic Surgery, Department of Surgery, Yale School of Medicine, New Haven, Connecticut, USA

While breast reconstruction (BR) remains a cornerstone of breast cancer care by restoring physical form and psychological well-being, safely extending these benefits to high-risk patients with increasingly complex comorbidities, particularly diabetes, remains a significant challenge^1^.

In this retrospective cohort study, the National Surgical Quality Improvement Program (NSQIP) (2021–2023) was queried to examine whether preoperative glycaemic control, quantified by haemoglobin A1c (HbA1c), was associated with 30-day postoperative morbidity following single-stage BR. NSQIP is a validated and risk-adjusted surgical quality improvement registry that aggregates outcomes data from hundreds of participating hospitals worldwide. The authors identified female breast cancer patients with diabetes and documented HbA1c undergoing single-stage autologous (ABR) or implant-based breast reconstruction (IBR) via International Classification of Diseases (ICD)/Current Procedural Terminology (CPT) codes^1–2^. Multistage procedures and incomplete records were excluded. Outcomes were assessed over the standardized 30-day postoperative period. Continuous and categorical variables were compared using the Student’s *t* test and χ^2^ analysis, respectively. Statistical significance was defined as a two-tailed *P* < 0.05.

Of the 739 patients meeting inclusion criteria, 606 (82%) underwent IBR and 133 (18%) underwent ABR. Patients were stratified according to the American Diabetes Association’s^3^ threshold for poor glycaemic control (HbA1c ≥ 6.5%). Among patients undergoing IBR, 55% exhibited HbA1c ≥ 6.5%. Baseline comorbidities, including ASA class, obesity, and hypertension, were comparable, apart from higher smoking prevalence in the high HbA1c group (*P* = 0.014). Notably, the composite 30-day complication rate—encompassing mortality, reoperation, readmission, and both surgical and medical complications—did not differ significantly between glycaemic control groups in the IBR cohort (15.0% *versus* 15.4%; *P* = 0.90). *Table 1* provides an overview of all extracted variables.

**Table 1:** Patient demographics, comorbidities, surgical characteristics, and outcomes following implant-based or autologous breast reconstruction (*n* = 739)

	Implant-based overall (*n* = 606)	Implant-based HbA_1c_ < 6.5 (*n* = 273)	Implant-based HbA_1c_ ≥ 6.5 (*n* = 333)	P*	Autologous overall (*n* = 133)	Autologous HbA_1c_ < 6.5 (*n* = 76)	Autologous HbA_1c_ ≥ 6.5 (*n* = 57)	*P*†
**Demographics**								
Age (years), mean(s.d.)	58(10)	57(10)	59(9.4)	0.162	58(9.6)	58(9.2)	58(10)	0.574
Height (in), mean(s.d.)	63(7.2)	63(10)	64(2.8)	0.347	60(24)	57(32)	63(2.8)	0.136
Weight (lbs), mean(s.d.)	184(40)	183(42)	185(38.5)	0.496	185(37)	187(40)	182(32)	0.484
BMI (kg/m2), mean(s.d.)	32(6.4)	32(6.7)	32(6.2)	0.155	32(6.1)	32(6.8)	32(5.3)	0.877
Gender				> 0.999				> 0.999
Female	606 (100%)	273 (100%)	333 (100%)		133 (100%)	76 (100%)	57 (100%)	
Ethnicity				0.842				0.362
Hispanic	93 (15.3%)	43 (15.8%)	50 (15.0%)		15 (11.3%)	6 (7.9%)	9 (15.8%)	
Race				0.339				0.238
White	339 (55.9%)	165 (60.4%)	174 (52.3%)		67 (50.4%)	41 (53.9%)	26 (45.6%)	
Black or African American	132 (21.8%)	51 (18.7%)	81 (24.3%)		32 (24.1%)	17 (22.4%)	15 (26.3%)	
Asian	48 (7.9%)	21 (7.7%)	27 (8.1%)		14 (10.5%)	10 (13.2%)	4 (7.0%)	
Native Hawaiian or Pacific Islander	4 (0.7%)	1 (0.4%)	3 (0.9%)		0 (0%)	0 (0%)	0 (0%)	
American Indian or Alaska Native	1 (0.2%)	0 (0%)	1 (0.3%)		0 (0%)	0 (0%)	0 (0%)	
Unknown/not reported	82 (13.5%)	35 (12.8%)	47 (14.1%)		20 (15.0%)	8 (10.5%)	12 (21.1%)	
**Preoperative health and comorbidities**								
Obesity (BMI ≥ 30 kg/m2)	343 (56.6%)	150 (54.9%)	193 (58.0%)	0.457	81 (60.9%)	47 (61.8%)	34 (59.6%)	0.798
Diabetes				< 0.001				0.004
Oral agents	459 (75.7%)	226 (82.8%)	233 (70.0%)		110 (82.7%)	69 (90.8%)	41 (71.9%)	
Insulin-treated	147 (24.3%)	47 (17.2%)	100 (30.0%)		23 (17.3%)	7 (9.2%)	16 (28.1%)	
Smoking	44 (7.3%)	12 (4.4%)	32 (9.6%)	0.014	6 (4.5%)	4 (5.3%)	2 (3.5%)	0.630
Ventilator dependence	0 (0%)	0 (0%)	0 (0%)	> 0.999	0 (0%)	0 (0%)	0 (0%)	> 0.999
COPD	9 (1.5%)	4 (1.5%)	5 (1.5%)	0.971	2 (1.5%)	2 (2.6%)	0 (0%)	0.217
Ascites	0 (0%)	0 (0%)	0 (0%)	> 0.99	0 (0%)	0 (0%)	0 (0%)	> 0.999
CHF	8 (1.3%)	1 (0.4%)	7 (2.1%)	0.063	1 (0.8%)	1 (1.3%)	0 (0%)	0.385
Hypertension	426 (70.3%)	183 (67.0%)	243 (73.0%)	0.111	84 (63.2%)	51 (67.1%)	33 (57.9%)	0.276
Renal failure	0 (0%)	0 (0%)	0 (0%)	> 0.999	0 (0%)	0 (0%)	0 (0%)	> 0.999
Dialysis	0 (0%)	0 (0%)	0 (0%)	> 0.999	0 (0%)	0 (0%)	0 (0%)	> 0.999
Corticosteroid use/immunosuppression	33 (5.4%)	12 (4.4%)	21 (6.3%)	0.302	5 (3.8%)	3 (3.9%)	2 (3.5%)	0.895
Bleeding disorder	10 (1.7%)	6 (2.2%)	4 (1.2%)	0.338	1 (0.8%)	1 (1.3%)	0 (0%)	0.385
Blood transfusions	0 (0%)	0 (0%)	0 (0%)	> 0.999	0 (0%)	0 (0%)	0 (0%)	> 0.999
History of sepsis	2 (0.3%)	1 (0.4%)	1 (0.3%)	0.888	0 (0%)	0 (0%)	0 (0%)	> 0.999
ASA Class				0.492				0.303
1. No disturbance	4 (0.7%)	3 (1.1%)	1 (0.3%)		0 (0%)	0 (0%)	0 (0%)	
2. Mild disturbance	220 (36.3%)	101 (37.0%)	119 (35.7%)		51 (38.3%)	32 (42.1%)	19 (33.3%)	
3. Severe disturbance	381 (62.9%)	169 (61.9%)	212 (63.7%)		82 (61.7%)	44 (57.9%)	38 (66.7%)	
4. Life-threatening	0 (0%)	0 (0%)	0 (0%)		0 (0%)	0 (0%)	0 (0%)	
Functional status				0.448				> 0.999
Independent	598 (98.7%)	271 (99.3%)	327 (98.2%)		133 (100%)	76 (100%)	57 (100%)	
Totally dependent	0 (0%)	0 (0%)	0 (0%)		0 (0%)	0 (0%)	0 (0%)	
Partially dependent	1 (0.2%)	0 (0%)	1 (0.3%)		0 (0%)	0 (0%)	0 (0%)	
Unknown	7 (1.2%)	2 (0.7%)	5 (1.5%)		0 (0%)	0 (0%)	0 (0%)	
**Surgical characteristics**								
Surgical specialty				0.089				0.193
General	336 (55.4%)	141 (51.6)	195 (58.6)		41 (30.8%)	20 (26.3%)	21 (36.8%)	
Plastic surgery	270 (44.6%)	132 (48.4%)	138 (41.4%)		92 (69.2%)	56 (73.7%)	36 (63.2%)	
Type of anaesthesia				> 0.999				> 0.999
General	606 (100%)	273 (100%)	333 (100%)		133 (100%)	76 (100%)	57 (100%)	
Flap type								
Free flap					105 (78.9%)	66 (86.8%)	39 (68.4%)	0.010
Latissimus dorsi flap					18 (13.5%)	6 (7.9%)	12 (21.1%)	0.028
TRAM flap					4 (3.0%)	2 (2.6%)	2 (3.5%)	> 0.999
Other					6 (4.5%)	2 (2.6%)	4 (7.0%)	0.401
Year of surgery				< 0.001				0.559
2021	207 (34.2%)	121 (44.3%)	86 (25.8%)		56 (42.1%)	35 (46.1%)	21 (36.8%)	
2022	192 (31.7%)	62 (22.7%)	130 (39.0%)		42 (31.6%)	22 (28.9%)	20 (35.1%)	
2023	207 (34.2%)	90 (33.0%)	117 (35.1%)		35 (26.3%)	19 (25.0%)	16 (28.1%)	
Setting				0.478				0.057
Inpatient	130 (21.5%)	55 (20.1%)	75 (22.5%)		115 (86.5%)	62 (81.6%)	53 (93.0%)	
Outpatient	476 (78.5%)	218 (79.9%)	258 (77.5%)		18 (13.5%)	14 (18.4%)	4 (7.0%)	
**30-Day postoperative outcomes**								
Length of hospital stay (days), mean(s.d.)	0.89(0.9)	0.85(0.87)	0.92(0.86)	0.332	3.3(1.5)	3.4(1.5)	3.0(1.4)	0.135
Operative time (minutes), mean(s.d.)	205(85)	203(91.2)	207(79.5)	0.517	425(176)	433(163)	413(193)	0.521
Any complication	92 (15.2%)	42 (15.4%)	50 (15.0%)	0.900	44 (33.1%)	19 (25.0%)	25 (43.9%)	0.022
Mortality	0 (0%)	0 (0%)	0 (0%)	> 0.999	0 (0%)	0 (0%)	0 (0%)	> 0.999
Reoperation	41 (6.8%)	21 (7.7%)	20 (6.0%)	< 0.001	11 (8.3%)	5 (6.6%)	6 (10.5%)	0.652
Readmission	26 (4.3%)	16 (5.9%)	10 (3.0%)	< 0.001	8 (6.0%)	2 (2.6%)	6 (10.5%)	0.187
Unplanned readmission	25 (4.1%)	16 (5.9%)	9 (2.7%)	0.213	8 (6.0%)	2 (2.6%)	6 (10.5%)	0.155
Surgical complication	56 (9.2%)	28 (10.3%)	28 (8.4%)	0.434	31 (23.3%)	10 (13.2%)	21 (36.8%)	0.001
Superficial incisional infection	18 (3.0%)	8 (2.9%)	10 (3.0%)	0.958	9 (6.8%)	4 (5.3%)	5 (8.8%)	0.425
Deep incisional infection	10 (1.7%)	5 (1.8%)	5 (1.5%)	0.751	2 (1.5%)	0 (0%)	2 (3.5%)	0.1
Organ space infection	19 (3.1%)	8 (2.9%)	11 (3.3%)	0.793	2 (1.5%)	0 (0%)	2 (3.5%)	0.1
Dehiscence	9 (1.5%)	7 (2.6%)	2 (0.6%)	0.047	6 (4.5%)	2 (2.6%)	4 (7.0%)	0.228
Bleeding requiring transfusion	4 (0.7%)	4 (1.5%)	0 (0%)	0.041	15 (11.3%)	5 (6.6%)	10 (17.5%)	0.089
Medical complication	12 (2.0%)	6 (2.2%)	6 (1.8%)	0.728	6 (4.5%)	3 (3.9%)	3 (5.3%)	> 0.999
Pneumonia	1 (0.2%)	0 (0%)	1 (0.3%)	0.365	0 (0%)	0 (0%)	0 (0%)	> 0.999
Reintubation	0 (0%)	0 (0%)	0 (0%)	> 0.999	0 (0%)	0 (0%)	0 (0%)	> 0.999
Pulmonary embolism	3 (0.5%)	1 (0.4%)	2 (0.6%)	0.683	1 (0.8%)	1 (1.3%)	0 (0%)	0.385
Ventilator >48 h	0 (0%)	0 (0%)	0 (0%)	> 0.999	0 (0%)	0 (0%)	0 (0%)	> 0.999
Cerebrovascular accident or stroke	1 (0.2%)	1 (0.4%)	0 (0%)	0.450	0 (0%)	0 (0%)	0 (0%)	> 0.999
Myocardial infarction	0 (0%)	0 (0%)	0 (0%)	> 0.99	0 (0%)	0 (0%)	0 (0%)	> 0.999
Cardiac arrest requiring CPR	1 (0.2%)	0 (0%)	0 (0%)	0.365	0 (0%)	0 (0%)	0 (0%)	> 0.999
Deep vein thrombosis/thrombophlebitis	2 (0.3%)	2 (0.7%)	0 (0%)	0.118	3 (2.3%)	2 (2.6%)	1 (1.8%)	0.736
Acute renal failure	0 (0%)	0 (0%)	0 (0%)	> 0.999	0 (0%)	0 (0%)	0 (0%)	> 0.999
Urinary tract infection	3 (0.5%)	3 (1.1%)	0 (0%)	0.055	1 (0.8%)	1 (1.3%)	0 (0%)	0.385
Septic shock	0 (0%)	0 (0%)	0 (0%)	> 0.99	0 (0%)	0 (0%)	0 (0%)	> 0.999
Sepsis	5 (0.8%)	3 (1.1%)	2 (0.6%)	0.500	2 (1.5%)	0 (0%)	2 (3.5%)	0.100
*Clostridium difficile* infection	0 (0%)	0 (0%)	0 (0%)	> 0.99	0 (0%)	0 (0%)	0 (0%)	> 0.999
**Discharge destination**				0.657				> 0.999
Home	603 (99.5%)	272 (99.6%)	331 (99.4%)		133 (100%)	76 (100%)	57 (100%)	
Not home	1 (0.2%)	0 (0%)	1 (0.3%)		0 (0%)	0 (0%)	0 (0%)	
Other/unknown	2 (0.3%)	1 (0.4%)	1 (0.3%)		0 (0%)	0 (0%)	0 (0%)	

Values are *n* (%) unless otherwise stated. HbA1c, haemoglobin A1c; s.d., standard deviation; BMI, body mass index; COPD, chronic obstructive pulmonary disease; CHF, congestive heart failure; ASA, American Society of Anesthesiologists; TRAM, transverse rectus abdominus myocutaneous; CPR, cardiopulmonary resuscitation. *P-values were calculated using Student's t test for continuous variables and chi-square test or Fisher's exact test for categorical variables (implant-based comparison). † *P*-values were calculated using Student's t test for continuous variables and chi-square test or Fisher's exact test for categorical variables (autologous comparison).

In contrast, outcomes diverged markedly in the ABR cohort. Of the 57 patients with HbA1c ≥ 6.5%, 43.9% experienced any complication, compared with 25.0% among those with HbA1c < 6.5% (*P* = 0.022). Flap selection also varied. Free flaps were more common in the control group (86.8% *versus* 68.4%; *P* = 0.010) and latissimus dorsi flaps more common in the high HbA1c group (21.1% *versus* 7.9%; *P* = 0.028).

This procedure-specific susceptibility of ABR to dysglycaemia may reflect the greater physiological burden of autologous procedures. ABR demonstrated significantly longer operative times than IBR (mean(standard deviation) 425(176) *versus* 205(85) minutes; *P* < 0.001)), which, alongside greater tissue trauma, amplifies metabolic stress. Under these conditions, poorly controlled diabetes may further compromise host defence, delay wound healing, and predispose to postoperative complications^2^. Conversely, the shorter duration and lower invasiveness of IBR may attenuate the impact of suboptimal glycaemic control on early outcomes^4^.

Nearly half of patients with HbA1c ≥ 6.5% who underwent ABR developed a complication within the first postoperative month, raising concerns about proceeding with flap procedures in this subgroup without adequate metabolic optimization. Although these data should be interpreted with appropriate caution, they highlight the potential importance of procedure-specific risk stratification rather than a ‘one-size-fits-all’ approach. IBR might represent a comparatively safer option when surgical timelines are urgent or glycaemic control remains suboptimal; however, further research is essential.

This study is limited by the 30-day NSQIP follow-up window, which precludes assessment of delayed complications like capsular contracture or late implant loss. Although the authors’ analysis did not identify an association between elevated HbA1c and early morbidity following single-stage implant-based reconstruction, Capito *et al*. ^5^ reported increased implant loss among patients with diabetes undergoing two-stage reconstruction, suggesting that glycaemic dysregulation may exert delayed effects not captured within the NSQIP window. In addition, the registry does not allow for complication grading. Furthermore, granular intraoperative or reconstructive details, such as implant positioning or flap perfusion parameters, were unavailable, limiting mechanistic insight. Future studies are warranted to further elucidate the relationship between glycaemic control and postoperative outcomes following breast reconstruction.

As breast cancer survivorship rises and type 2 diabetes becomes increasingly prevalent, reconstructive surgeons will be challenged to navigate complex risk profiles with limited evidence-based guidance. The present study represents a step toward individualized, data-driven surgical decision-making by identifying potential procedure-specific metabolic vulnerabilities.

## Data Availability

Data sets generated and analyzed to provide the findings in this study are available from the corresponding author upon reasonable request. The data are not publicly available due to restrictions from the American College of Surgeons National Surgical Quality Improvement Program.

## References

[1] Knoedler S, Kauke-Navarro M, Knoedler L, Friedrich S, Ayyala HS, Haug V et al The significance of timing in breast reconstruction after mastectomy: an ACS-NSQIP analysis. J Plast Reconstr Aesthet Surg 2024;89:40–50 38134626 10.1016/j.bjps.2023.11.049

[2] Panayi AC, Knoedler S, Alfertshofer M, Matar DY, Friedrich S, Rühl J et al The relevance of optimal preoperative glycemic control for outcomes of patients with diabetes undergoing surgery. Ann Surg 2025;9900:10.1097/SLA.0000000000006664 10.1097/SLA.0000000000006664 39936298

[3] American Diabetes Association Professional Practice Committee . 2. Diagnosis and classification of diabetes: standards of care in diabetes—2025. Diabetes Care 2024;48:S27–S49 10.2337/dc25-S002 PMC11635041 39651986

[4] Rocco N, Catanuto GF, Accardo G, Velotti N, Chiodini P, Cinquini M et al Implants versus autologous tissue flaps for breast reconstruction following mastectomy. Cochrane Database Syst Rev 2024;10:Cd013821 39479986 10.1002/14651858.CD013821.pub2 PMC11526434

[5] Capito AE, Potturi N, Canzoneri CN, Applebaum MA, Hamlin S, Choi J et al Effects of screening hemoglobin A1C on complications in implant-based breast reconstruction. Eplasty 2024;24:e63 40463921 PMC12132407

